# Characterisation of alternative expression vectors for recombinant Bacillus Calmette-Guérin as live bacterial delivery systems

**DOI:** 10.1590/0074-02760190347

**Published:** 2020-05-15

**Authors:** Larissa V Nascimento, Carina C Santos, Luciana CC Leite, Ivan P Nascimento

**Affiliations:** 1 Instituto Butantan, Laboratório Especial de Desenvolvimento de Vacinas, São Paulo, SP, Brasil; 2 Universidade de São Paulo, Programa de Pós-Graduação Interunidades em Biotecnologia, São Paulo, SP, Brasil; 3 Universidade Federal da Bahia, Faculdade de Farmácia, Departamento de Análises Clínicas e Toxicológicas, Salvador, BA, Brasil

**Keywords:** recombinant BCG, expression vectors, promoters, GFP

## Abstract

**BACKGROUND:**

Bacillus Calmette-Guérin (BCG) is considered a promising live bacterial delivery system. However, several proposals for rBCG vaccines have not progressed, mainly due to the limitations of the available expression systems.

**OBJECTIVES:**

To obtain a set of mycobacterial vectors using a range of promoters with different strengths based on a standard backbone, previously shown to be stable.

**METHODS:**

Mycobacterial expression vectors based on the pLA71 vector as backbone, were obtained inserting different promoters (P_AN_, P_αAg,_ P_Hsp60_, P_BlaF*_ and P_L5_) and the green fluorescence protein (GFP) as reporter gene, to evaluate features such as their relative strengths, and the *in vitro* (inside macrophages) and *in vivo* stability.

**FINDINGS:**

The relative fluorescence observed with the different vectors showed increasing strength of the promoters: P_AN_ was the weakest in both *Mycobacterium smegmatis* and BCG and P_BlaF*_ was higher than P_Hsp60_ in BCG. The relative fluorescence observed in a macrophage cell line showed that P_BlaF*_ and P_Hsp60_ were comparable. It was not possible to obtain strains transformed with the extrachromosomal expression vector containing the P_L5_ in either species.

**MAIN CONCLUSION:**

We have obtained a set of potentially stable mycobacterial vectors with a arrange of expression levels, to be used in the development of rBCG vaccines.

The Bacillus Calmette-Guerín (BCG) is the only licensed vaccine against tuberculosis. It is an attenuated strain of *Mycobacterium bovis* obtained after successive passages in glycerol-potato medium for about 15 years, from 1906 to 1920.^1^ Since its introduction in 1921, it has been administered to more than three billion individuals with few reported side effects, being one of the safest vaccines in the world.^1^ In addition, features such as low production cost, heat stability, induction of long-lasting type 1 helper T cell (Th1) immunity and potent immunostimulation, have motivated its investigation as a live delivery vector for the development of new vaccines against important infectious diseases.^2^ ^,^ ^3^ ^,^ ^4^ ^,^ ^5^ ^,^ ^6^ ^,^ ^7^ ^,^ ^8^ BCG is also the most successful immunotherapeutic agent for treating non-muscle invasive bladder cancer.^9^ In addition, there are studies implicating BCG vaccination as responsible for non-specific protection against other infectious diseases, also called trained immunity or off-target effects.^10^

Pioneering research in the genetic manipulation of *Mycobacterium*, such as performed by Jacobs Jr et al. allowed the introduction of foreign DNA into *Mycobacterium smegmatis* and BCG.^11^ This opened the possibility for development of multi-vaccines based on BCG as a live vehicle to present heterologous antigens from different pathogens. Since then, several groups have had success in the development of rBCG strains, expressing antigens from virus, bacteria or parasites, or even cytokine molecules.^1^ ^,^ ^2^ ^,^ ^12^ Despite several reports on the successful construction of rBCG strains, many rBCG constructs did not attain expression, revealing the importance of characterising other expression vectors construct. On the other hand, there are studies that have indicated a role for antigen expression level in the induction of immune responses. For the *Mycobacterium tuberculosis* (*Mtb*) antigen, 85B, low-level expression skewed the immune response towards a T helper 1 (Th1) immune response, and higher expression levels towards a Th2 immune response.^13^

Different features can influence antigen expression in BCG, such as promoter strength, codon usage, vector stability, as well as the strain of BCG. Although, all these characteristics together can influence gene expression, the promoter is surely the key factor of this system.^14^ ^,^ ^15^

Several promoters have been used to express a variety of genes in mycobacteria. For some time, the heat shock promoter, P_Hsp60_, was the preferential choice, in part for being considered a strong promoter.^15^ However, several other genes have not been expressed yet, even using strong promoters such as P_Hsp60_. The use of other promoters was described, such as the mutated beta-lactamase promoter, P_BlaF*_ (from *M. fortuitum*),^12^ the alpha antigen promoter, P_αAg_ (from BCG), the 19 kDa antigen promoter (from *Mtb*) or the *M. paratuberculosis* P_AN_ promoter (Table), being P_BlaF*_ considered the stronger and P_AN_ the weaker when compared between them. As consequence, this allowed gene expression at different levels in mycobacteria. The strength of different promoters has been evaluated,^15^ ^,^ ^16^ with different reporter genes and plasmids as backbone, making it difficult to compare the studies.

Another feature that is important for antigen expression is vector stability. We have constructed several rBCG strains using the pLA71 plasmid^12^ ^,^ ^17^ as a backbone vector, expressing a genetically detoxified subunit 1, S1PT,^6^ CRM197^18^ and tetanus toxin fragment C.^19^ Especially for the rBCG-S1PT construct, we have observed high stability *in vivo*.^6^ On the other hand, it has been shown that maintaining the plasmid based on antibiotic resistance does not determine that the antigen will be expressed over time.^20^ Rizze et al.^20^ developed an auxotrophic mycobacterial system based on *leuD* complementation to express 85B and 85BT antigens (BCG *ΔleuD*-85B and BCG *ΔleuD*-85BT), which proved to be stable both for the presence of the plasmid and for the expression of these antigens over a long period of time after immunisation of mice. We have previously obtained a BCG strain auxotrophic for lysine, (BCG-*Δ-lysA*), complemented with a pLA71-based expression vector^7^ which was shown to maintain expression of the antigen through several *in vitro* passages without antibiotic selection (results not shown). These results indicate the stability of the pLA71-based backbone vectors.

Therefore, considering that it is still important to optimise expression systems for the development of new rBCG strains,^15^ the aim of the current work was to obtain a collection of mycobacterial vectors based on a vector backbone previously tested for stability, using a range of promoters with different strengths. The activity of the different promoters was evaluated using the green fluorescent protein (GFP) as reporter for gene expression. *In vitro* and *in vivo* stability was also evaluated.

## MATERIALS AND METHODS

*Strains* - *Escherichia coli* DH5a, *M. smegmatis* mc^2^ 155 and *M. bovis* BCG strain Moreauwere used in this study. *E. coli* was grown in Luria-Bertani medium at 37ºC. The *M. smegmatis* derived strains were grown in Middlebrook 7H9 (Difco Laboratories, Detroit, MI) or Middlebrook 7H10 agar (Difco Laboratories) with 0.5% glycerol and 0.05% Tween 80 (MB7H9 and MB7H10, respectively), plus kanamycin (20 µg/mL) when required, at 37ºC in a humidified 5% CO_2_ incubator.

BCG or the constructs, were grown in MB7H9 or MB7H10 agar with 10% OADC, 0.5% glycerol and 0.05% Tween 80, plus kanamycin (20 µg/mL) when required, at 37ºC in a humidified 5% CO_2_ incubator, until cultures reached an optical density (OD) of ~ 0.8. Bacteria were harvested by centrifugation at 2.800 x g, washed twice with distilled water and resuspended in 10% Glycerol. The mycobacterial preparations were maintained at -80ºC until used and Colony-forming units (CFU) were determined after 48 h.

*Construction of the vectors* - The *gfp* gene was amplified from the pEGFP-N1 vector (Clontech Laboratories), including a *Kpn I* restriction site at the 5’ end and a *Not I* restriction site at the 3’ end [Supplementary data **(Table)**]. The promoters P_Hsp60_ and P_αAg_ were amplified by polymerase chain reaction (PCR) using genomic DNA isolated from BCG as template. The P_BlaF*_, P_AN_ and P_L5_ promoters were amplified by PCR from plasmids containing the sequences, previously used in our lab.^5^ ^,^ ^6^ ^,^ ^8^ ^,^ ^14^

A *BamH I* restriction site was included at the 5’ end and a *Kpn I* restriction site was included at the 3’ end of the promoters [Supplementary data **(Table)**]. These fragments, promoters and *gfp*, were fused by Double-joint PCR technique, according to Yu et al.^21^ The expression cassettes were formed and cloned into the pLA71 vector at the *BamH I* and *Not I* sites. DH5α *E. coli* transformed with the expression vectors resulted in colonies that were screened by colony PCR for the presence of the respective fusion gene inserts. DNA from the generated plasmids (pNN71-pΣ) were extracted and the correct constructs were confirmed by sequencing. pJH223-P_L5_-*gfp*, is derived from an integrative vector, pJH223, and kindly provided by Dr William Jacobs (Albert Einstein College of Medicine - NY, USA).

*Transformation of mycobacteria* - Aliquots (100 μL) of competent mycobacterial cells were electroporated with 1-2 μg of DNA with a single pulse (2.5 kV; 25 μF; 600 ohms) using a Bio-Rad Gene Pulser. Transformed *M. smegmatis* cells were incubated in 4 mL MB7H9 medium at 37ºC for 3 h; transformed BCG was incubated under the same conditions for 24 h, before plating in MB7H10 containing kanamycin (20 µg/mL). Plates were incubated at 37ºC in a humidified 5% CO_2_ incubator: *M. smegmatis* for three days and three weeks for BCG. Individual clones were cultivated in MB7H9, washed and stored frozen in phosphate buffered saline (PBS) and 10% glycerol at -80ºC until used. Individual mycobacterial colonies were analysed directly using a Nikon Eclipse E200 fluorescence microscope. Transformation of BCG with these vectors generated the following constructs: rBCG-pLA71-P_αAg_ *-gfp*, rBCG-pLA71-P_AN_-*gfp*, rBCG-pLA71-P_Hsp60_-*gfp*and BCG-pJH223-P_*L5*_ -*gfp*.

*Flow cytometric analysis of fluorescent bacteria* - To measure the fluorescence in the mycobacterial transformants expressing GFP, samples were thawed and resuspended in PBS. The geometric mean fluorescence was determined for the bacterial populations by flow cytometry using a FACSCanto II (BD Biosciences).

*Infection of a macrophage cell line by the rBCG-pNN71-pΣstrains and confocal microscopy analysis* - The RAW 264.7 macrophage cell line was gently provided by Dr Maria Cristina Breno, from the Laboratory of Pharmacology, Institute Butantan. These cells were grown in Roswell Park Memorial Institute (RPMI) (Gibco-BRL) supplemented with 10% heat-inactivated foetal calf serum (FCS) (Gibco-BRL) at 37ºC in a humidified 5% CO_2_ incubator. The cells were seeded in 24-well plates (Corning) (1 x 10^6^ cells per well) and allowed to adhere overnight. The macrophages were then incubated with the mycobacterial suspensions at a multiplicity of infection (MOI) 1:10. After 3 h of infection, macrophages were washed to remove the remaining extracellular bacteria and the cells were incubated for an additional 24 h in fresh medium. The preparation of samples for confocal analysis was made as follow: cells were adhered to glass slides coated with poly-_L_-lysine (Sigma-Aldrich), fixed in 4% paraformaldehyde and quenched with 0.1 M glycine. Mounted slides were analysed with a confocal laser microscope system (Zeiss LSM 510).

*Immunisation of mice with rBCG-GFP strains and recovery of mycobacteria from the spleens* - Five female BALB/c mice, five to seven weeks, were inoculated i.p. with the rBCG-pLA71-P_AN_-*gfp* or rBCG-pLA71-P_Hsp60_-*gfp* strains at a concentration of 1 x 10^6^ CFU/ animal. After 30 days, the spleens were extracted, homogenised and serially diluted to be plated with (+) or without (-) kanamycin (Kan) for determination of colony forming units (CFU). Expression of GFP in the colonies were confirmed by fluorescence microscopy in all clones obtained. The bars show the mean plus the standard deviation of five animals per experiment.

*Statistical analysis* - Statistical analysis between groups were performed using Turkey test. The level of statistical significance was set at p < 0.001.

*Ethics* - All animal experiments were performed according to Brazilian and international guidelines on animal experimentation and approved by the Ethics Committee of Instituto Butantan, São Paulo-SP (CEUAIB), (Permit number 1264/14).

## RESULTS

**Fig. 1:**
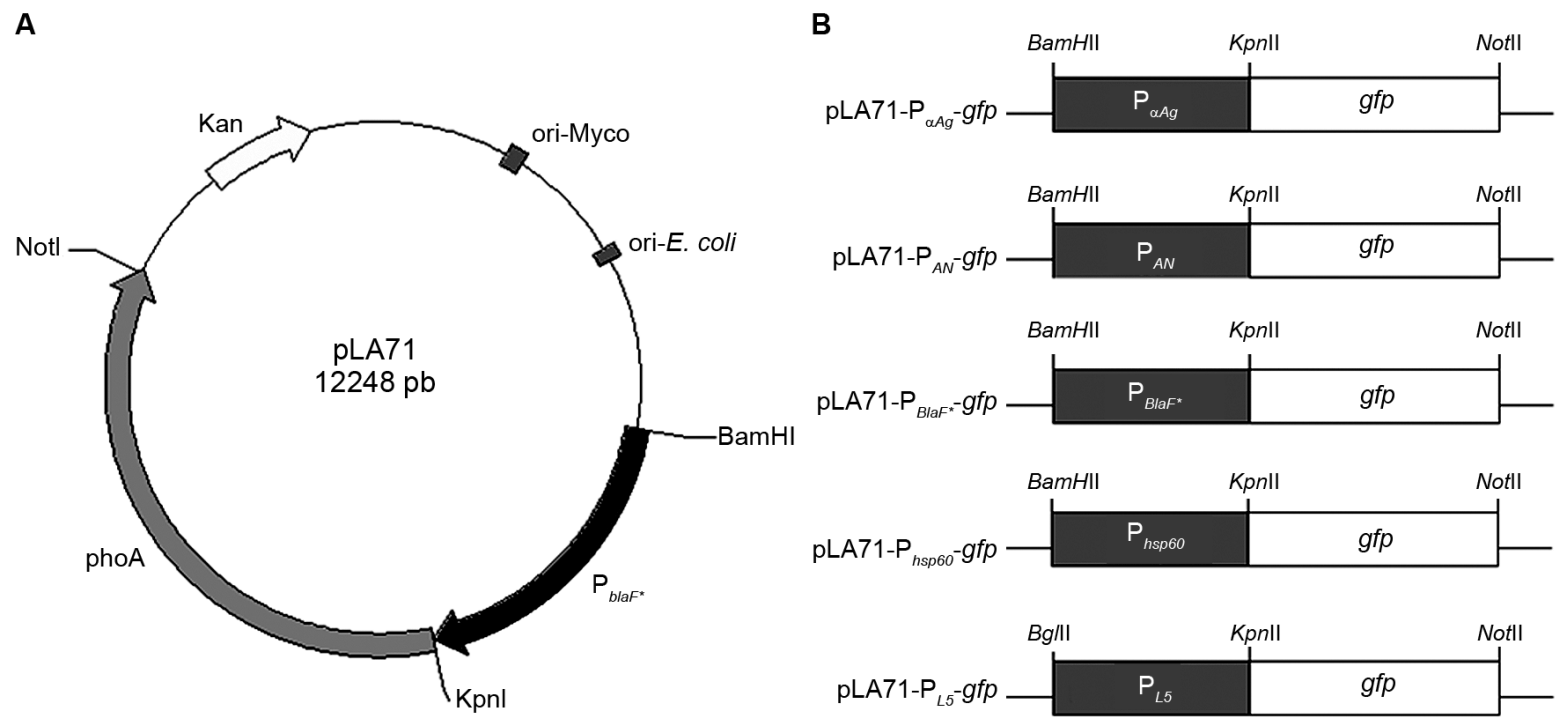
schematic representation of the pLA71 vector and the expression cassettes formed by the promoters and the *gfp* reporter gene. (A) pLA71 contains the β-lactamase promoter (P_BlaF*_),the kanamycin resistance gene (Kan), the *Escherichia coli* origin of replication (ori-*E. coli*) and mycobacterial origin of replication (ori-Myco), and the alkaline phosphatase gene (phoA). (B) The expression cassettes constructed using the mycobacterial promoters and the *gfp* gene by polymerase chain reaction (PCR).

*Construction of mycobacterial expression vectors containing promoters with different strengths using the pLA71 vector system* - To compare the strength of different promoters in mycobacteria, in a same vector backbone, we cloned known promoter sequences (Table) into the pLA71 vector system, placing the *gfp* gene under the control of each promoter (Fig. 1). An expression vector containing the recently characterised promoter from mycobacteriophage L5 (P_L5_),^14^ was also constructed using the same strategy. The different constructs were used to transform *M. smegmatis* and BCG and the transformants were selected initially by kanamycin resistance and then by GFP expression observed in a fluorescence microscope. Only colonies that showed fluorescence in the plates were expanded in liquid medium to be analysed by flow cytometry. It was possible to obtain clones for all constructs in *M. smegmatis* and BCG, except for the pLA71-P_L5_-*gfp* vector. Interestingly, the *E. coli* colonies transformed with the pLA71-P_L5_-*gfp* vector displayed a green phenotype, indicating that the construct was functional. Although toxicity of GFP has not been previously described,^14^ ^,^ ^22^ ^,^ ^23^ ^,^ ^24^ it is possible that a higher expression of GFP would be toxic to the mycobacteria.

**TABLE:** General features of the promoter used in this study

Promoter	Strength	Source	Reference
P_AN_	Low	*Mycobacterium paratuberculosis*	Murray et al.^30^
P_Hsp60_	High	Bacillus Calmette-Guérin (BCG)	Stover et al*.* ^(2)^
P_αAg_	Moderate	*M. kansassi*	Matsuo et al.^31^
P_BlaF*_	High	*M. fortuitum*	Timm et al.^(12)^
P_Left_	ND	Micobacteriophage L5	Nesbit et al.^32^

ND: not determined until now.

**Fig. 2:**
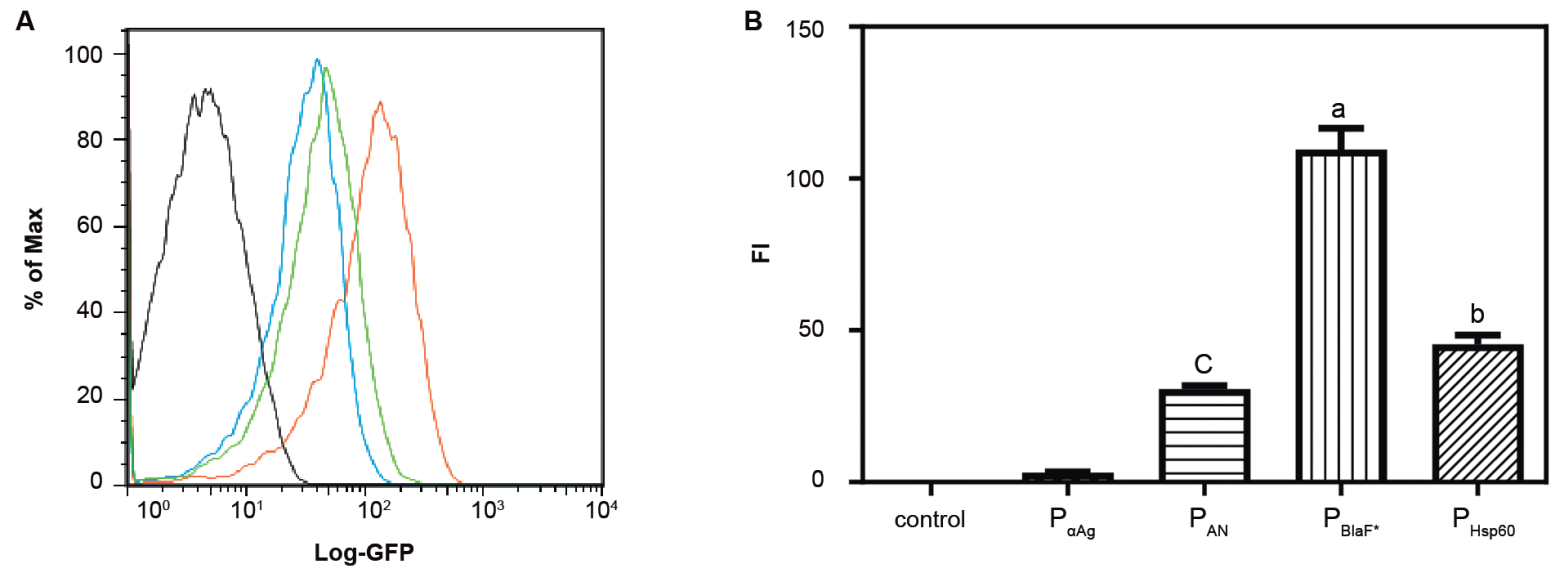
expression of *gfp* in *Mycobacterium smegmatis* transformed with the pNN71-pΣ plasmids. (A) Fluorescence histogram obtained from the rSmeg strains containing the pLA71 vectors with the different promoters: Grey, wtSmeg control (not visible); black, pLA71-P_αAg_-*gfp*; blue, pLA71-P_AN_-*gfp*; green, pLA71-P_Hsp60_-*gfp*; orange, pLA71-P_BlaF*_-*gfp*. (B) Total fluorescence obtained from the rSmeg strains expressing GFP through the different promoters. Results are mean ± standard deviation (SD). of five colonies from each construct in rSmeg. Differences were considered statistically significant when p ≤ 0.001: (a) as compared to all groups; (b) as compared to the P_AN_ group and (c) as compared to the wtSmeg control and P_αAg_ (one-way ANOVA).

*Differential fluorescence of the recombinant M. smegmatis strains containing the different expression vector systems* - The expression of GFP by the different constructs in *M. smegmatis* was evaluated by flow cytometry. Two or more colonies from each recombinant *M. smegmatis* (rSmeg) transformants were expanded in liquid medium and washed in PBS before analysis by flow cytometry. It was possible to observe that the different expression systems presented a variety of fluorescence intensities (Fig. 2). The expression vector based on the P_aAg_ promoter displayed very low fluorescence in the pLA71 background, comparable to wtBCG. The P_AN_ and the P_Hsp60_ promoters displayed intermediate fluorescence intensity and the P_BlaF*_ promoter was shown to be the strongest.

**Fig. 3:**
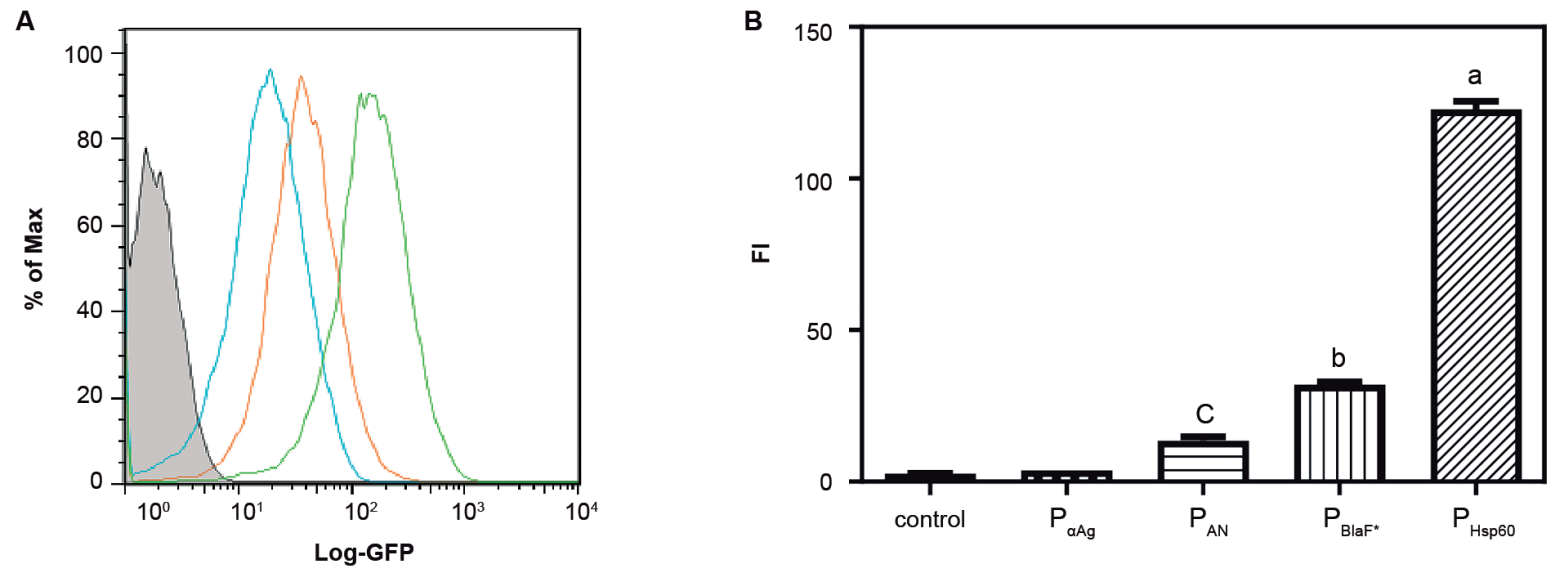
expression of *gfp* in *Mycobacterium bovis* Bacillus Calmette-Guérin (BCG) transformed with the pNN71-pΣ plasmids. (A) Fluorescence histogram obtained from the rBCG strains containing the pLA71 vectors with the different promoters: grey, wtBCG control; black, pLA71-P_αAg_-*gfp*(not visible); blue, pLA71-P_AN_-*gfp*; green, pLA71-P_Hsp60_-*gfp*; orange, pLA71-P_BlaF*_-*gfp*. (B) Total fluorescence obtained from the rBCG strains expressing GFP through the different promoters. Results are mean ± standard deviation (SD) of five colonies from each construct in rBCG. Differences were considered statistically significant when p ≤ 0.001: (a) as compared to all groups; (b) as compared to the P_AN_ group; and(c) as compared to the wtBCG control and P_αAg_ (one-way ANOVA).

**Fig. 4:**
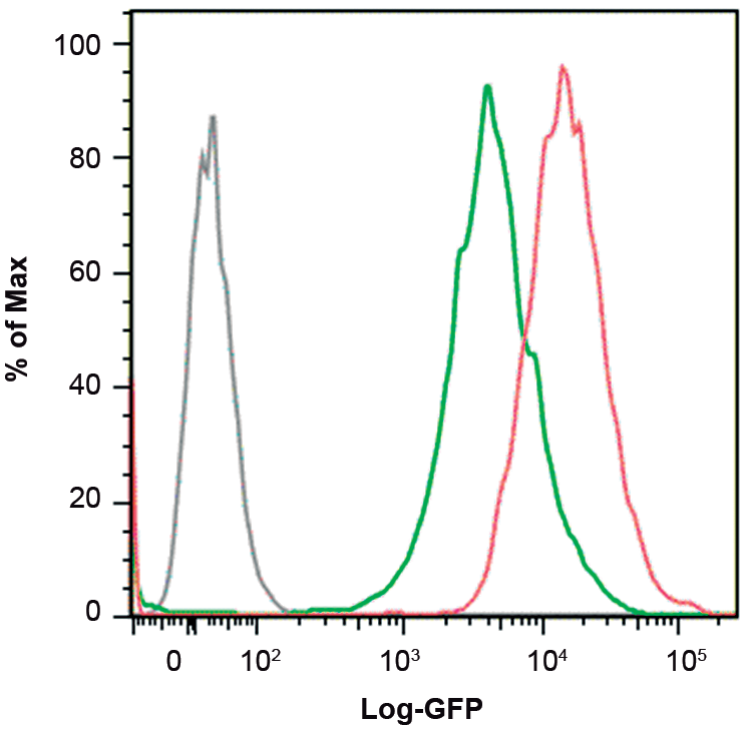
expression of green fluorescence protein (GFP) in Bacillus Calmette-Guérin (BCG) transformed with the pLA71-P_Hsp60_-*gfp* plasmid or the pJH223-P_L5_-*gfp* integrative vector. Histogram of fluorescence obtained from rBCG transformed with the vectors. The expression levels in rBCG were detected using flow cytometry. Grey, wtBCG control; green, rBCG-pLA71-P_Hsp60_-*gfp*; and red, rBCG-pJH223-P_L5_-*gfp*.

*Differential fluorescence of the recombinant BCG strains containing the different expression vector systems* - Analysing the same vectors in the BCG host, the expression based on the P_aAg_ promoter again displayed no significant fluorescence when compared to the wtBCG control. However, in this host, the P_AN_ and the P_BlaF*_ promoters displayed intermediate fluorescence intensities and the P_Hsp60_ promoter was shown to be the strongest (Fig. 3). The range of fluorescence obtained in the rBCG strains was comparable to that obtained inrSmeg strains (Figs 2-3). On the other hand, as was not possible to obtain rBCG transformants using the pLA71-P_L5_-*gfp* expression vector (that is using the P_L5_ promoter in the pLA71 backbone), we decided to use the integrative vector pJH223-P_*L5*_ -*gfp* containing the P_L5_ promoter previously characterised to verify and compare the expression level of GFP in rBCG with the pLA71-P_Hsp60_-*gfp* vector (Fig. 4).

**Fig. 5:**
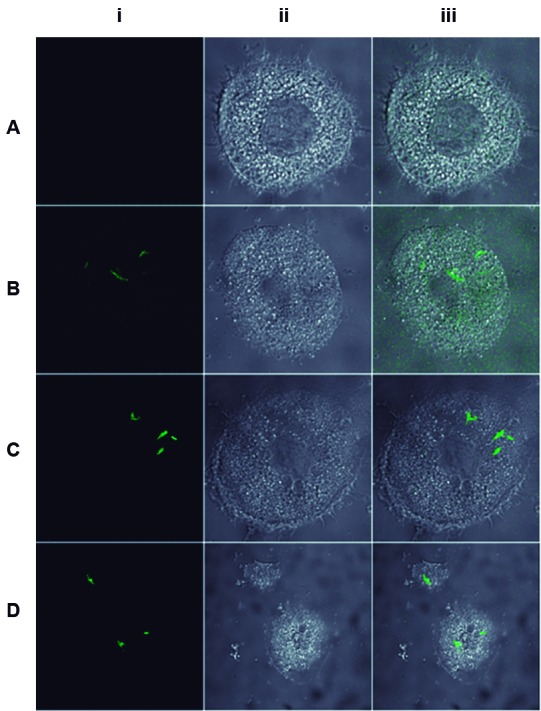
expression of the different rBacillus Calmette-Guérin-green fluorescence protein (rBCG-GFP) strains in the macrophage cell line, RAW 264.7. Confocal microscopy analysis of GFP expression in rBCG-pLA71-P_AN_-*gfp* (B), rBCG-pLA71-P_BlaF*_-*gfp* (C) and rBCG-pLA71-P_Hsp60_-*gfp* (D) inside the macrophage cell line, RAW 264.7. wtBCG was used as negative control (A). (i) FITC, (ii) Visible and (iii) FITC plus visible.

*Analyses of GFP expression in macrophages* - To investigate the activity of each promoter *in vitro*, the macrophage RAW 264.7 cell line was infected with the different rBCG constructs and analysed by confocal microscopy. GFP fluorescence can be observed from inside the macrophages with all rBCG strains (Fig. 5). The rBCG-pLA71-P_AN_-*gfp* displayed lower fluorescence then rBCG-pLA71-P_BlaF*_-*gfp* and rBCG-pLA71-P_Hsp60_-*gfp*, and the difference between the latter two was not so evident.

**Fig. 6:**
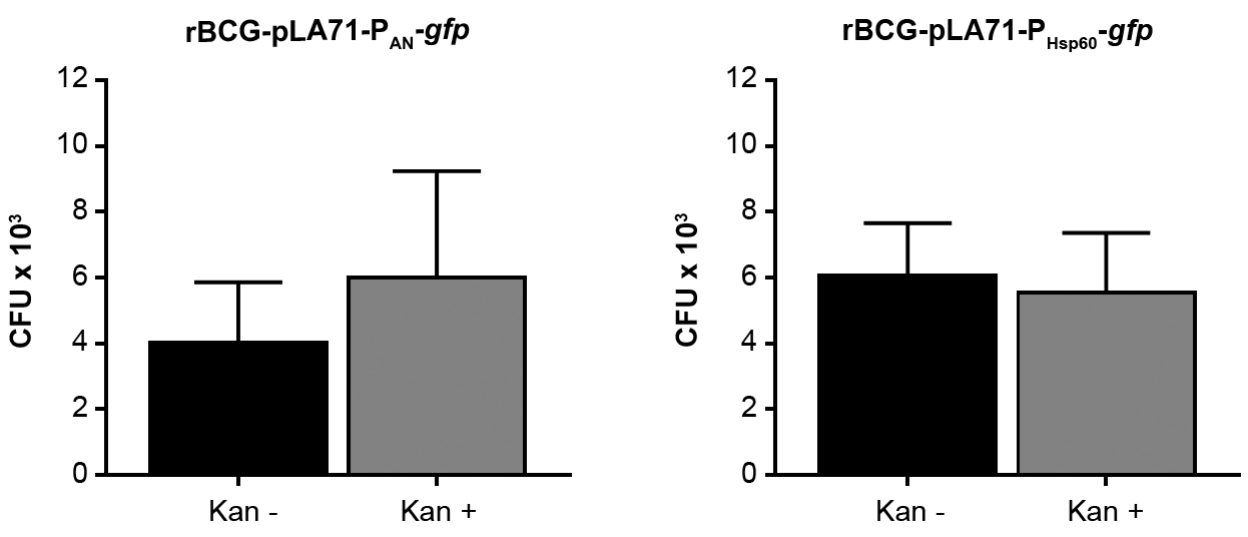
comparable stability of the vectors expressing high and low levels of green fluorescence protein (GFP) *in vivo*. Five female BALB/c mice, five to seven weeks, were inoculated i.p. with the rBCG-pLA71-P_AN_-*gfp* and rBCG-pLA71-P_Hsp60_-*gfp* strains at a concentration of 1 x 10^6^ colony forming units (CFU)/animal. After 30 days, the spleens were extracted, homogenised and serially diluted to be plated with (+) or without (-) kanamycin (Kan) for determination of CFU. The bars show the mean plus the standard deviation of five animals per experiment.

*Shuttle vectors stability in vivo* - In order to determine the *in vivo* vector stability of the rBCG-GFP constructs, the strains displaying the lowest and the highest expression levels were used, rBCG-pLA71-P_AN_-*gfp* and rBCG-pLA71-P_Hsp60_-*gfp*, respectively. BALC/c mice were inoculated i.p. with the rBCG strains and the mycobacteria were recovered from the spleens 30 days later and plated in the presence and absence of kanamycin. It can be observed that there is no difference between the number of bacteria recovered from the two strains, indicating that they have comparable stability (Fig. 6). Furthermore, the presence of kanamycin did not decrease the proportion of bacteria, indicating that the vector was relatively stable *in vivo*, that is, in the absence of antibiotic pressure. Furthermore, it could be observed that all the colonies recovered from the plates containing kanamycin displayed fluorescence.

## DISCUSSION

BCG has been used as a vaccine against tuberculosis for almost a century, being the most widely used vaccine in the world.^15^ It is a potent modulator of the immune response, which makes it a very attractive live vehicle for the presentation of heterologous antigens. However, important issues such as vector stability and antigen expression levels have been shown to play major roles in the success of different constructs of recombinant BCG vaccines. Most of the replicative vectors for mycobacteria were obtained using the pLA5000 origin of replication, which allows three to ten plasmid copies per bacterial cell.^12^ ^,^ ^25^ On the other hand, the level of expression of heterologous genes in mycobacteria is modulated mainly by the promoter, yet not solely. Although, there are several studies performed with a set of promoters and in different vectors as backbone,^20^ ^,^ ^26^ investigations in the search for new and improved promoters continue to be performed.^25^ ^,^ ^27^

We have extensive experience with the mycobacterial expression vector pLA71, containing the P_BlaF*_ promoter, which we have used to express many different antigens from different pathogens. Our experience has shown that this vector backbone is considerably stable *in vitro* and *in vivo,* including when used in an auxotrophic complementation strategy.^7^ Therefore, this vector backbone would be convenient to be used in future constructs. On the other hand, vaccines for different pathogens can require different antigen expression levels to achieve induction of appropriate immune responses. Therefore, it is important to compare promoters with different strengths in a same backbone shown to be stable.

In the present study, we have constructed a set of vectors based on the pLA71 backbone, containing a selection of promoters covering a range of expression strengths. In addition to these traditional promoters, we also evaluated a mycobacteriophage promoter, P_L5_ recently studied in our laboratory, considered strong, but never compared with others in the same study.^14^

We investigated the expression levels of the promoters considered to be weak, moderate and strong (Table) in the pLA71 background and in two species of mycobacteria, *M. smegmatis* and BCG. Our results showed that the P_AN_ promoter, which is considered the weakest among those evaluated, was also the weakest in both species, indicating that this promoter shows consistent results in different backgrounds and across species. However, the P_aAg_ promoter, considered to be moderate, did not present significant fluorescence in either species when compared to BCG control. Furthermore, it was also not possible to visualise green colonies under the fluorescence microscope (data not shown). It is possible that this promoter suffers some negative influence in the pLA71 background.

Furthermore, between the promoters considered to be strong (P_BlaF*_ and P_Hsp60_), the difference between them was shown to be dependent on the species, since P_BlaF*_ was shown to be stronger in *M. smegmatis* and P_Hsp60_ stronger in BCG (as observed *in vitro* in liquid medium). However, when we tested recombinant BCG strains in a macrophage infection assay and visualised them by confocal microscopy, strains driving expression of GFP by P_BlaF*_ and P_Hsp60_ seem not to be different, suggesting that the regulatory mechanisms for these two promoters can be influenced by the environment.

When analysing the same promoter in both *Mycobacterium* species we can observe that P_BlaF*_ induced fluorescence activity about 3-fold higher in *M. smegmatis* than in BCG. On the contrary, for P_Hsp60_, the fluorescence activity was about 3-fold higher in BCG than in *M. smegmatis*. In a similar study, using the P_Hsp60_ promoter and β-galactosidase activity as reporter in *M. smegmatis* and BCG under the same culture conditions, Dellagostin et al. observed a higher expression level in *M. smegmatis*.^26^ This contrasts with our results, but it is important to consider that these experiments used different reporter proteins, suggesting the importance of the gene sequence in the expression levels obtained.

P_L5_ is considered a strong promoter, however, it has not been compared to other promoters in a common expression vector for mycobacteria. We have previously investigated the original promoter P_L5_ cloned into the pJH152 vector for expression of GFP and submitted to randomised mutation through error-prone PCR. The obtained P_L5_ mutant promoters were compared seeking the higher or lower GFP expression levels in BCG.^14^ We could not compare our construct pLA71-pL5-*gfp* to the pJH152-pL5-*gfp* because our construct does not generate *M. smegmatis* or BCG transformants, although its functionality was demonstrated by high expression of GFP in *E. coli* clones. Perhaps, the expression level obtained by Kanno et al.^14^ is not high enough to be toxic, unlike ours. Furthermore, in a recent work, Oliveira et al.^28^ have compared several different promoters using the pUP500 vector as a backbone and the eGFP as reporter protein. However, although this work has the same idea, the purpose for Oliveira et al.^28^ was to employ the Biobrick strategy to construct a toolbox of several mycobacterial vector parts, including promoters and reporter genes. In fact, similar to our work, it is an effort to standardise a system that can enhance the efficacy and use of recombinant BCG.

Jain et al.^29^ measured the GFP expression by mycobacteriophages within mycobacteria using P_L5_ phage promoter in comparison with that obtained by the P_Hsp60_ promoter in the same system. They showed that the fluorescence activity was almost 100-fold higher in the first. Interestingly, transformants of pLA71-P_L5_-*gfp* could not be selected in either mycobacterial species, although the plasmid was functional in *E. coli* since we could observe green colonies (data not shown). It is possible that this can be due to the combination of the high strength of the promoter and the plasmid copy number (up to ten copies). The higher expression level may produce toxic amounts of the protein for the mycobacteria. When we used the integrative plasmid, pJH223-*gfp* (which has only one copy), fluorescence activity was higher than with P_Hsp60_, showing the strongest expression level within the rBCG strains studied (Fig. 4). Integrative vectors are known to allow greater stability but lower expression levels. Once transformants of pLA71-P_L5_-*gfp* were not obtained, it was not possible to compare the stability between episomal and integrative vectors in this study. However, greater levels of GFP expression were observed using pJH223-P_L5_-*gfp* vector, and even with only one copy, it shows higher expression levels that an episomal vector containing P_Hsp60_ for instance.

We have obtained a series of vectors to be used in the development of rBCG strains with previously determined level of antigen expression and vector stability, that can lead to more successful outcomes in rBCG vaccines.
