# Supplementary material for: Characterisation of alternative expression vectors for recombinant Bacillus Calmette-Guérin as live bacterial delivery systems
Source: Mem Inst Oswaldo Cruz. 2020 May 15;115:e190347. doi: 10.1590/0074-02760190347 (PMC7227789; doi:10.1590/0074-02760190347)
Supplement: Supplementary file 1 [file 1678-8060-mioc-115-e190347-s.pdf]

TABLE

Primers used for the constructs based on pLA71 vector

	Primers	Restriction sites[a]
gfp[b]	Forward: 5'-TAGTAGCATATGGTGAGC-AAGGGCGAGGA-3'<br>Reverse: 5'-TAGTAGCAGCTGTTACTTGTACAGCTCGTCCA-TGC-3'	*Nde*I<br>*Pvu*II
gfp[c]	Forward: 5'-ACCGCGGGCGCGGGTACCATGGTGAGCAAGGGC-3'<br>Reverse: 5'-TAGGCGGCCGCTTACTTGTACAGCTCGTCCATGC-3'	*Kpn*I<br>*Not*I
$P_{αAg}$	Forward: 5'-TAGGGATCCACGACTTTCGCCCGAATCGA-3'<br>Reverse: 5'-GCCCTTGCTCACCATGGTACCCGCGCCCGCGGT-3'	*BamH*I<br>*Kpn*I
P_{AN}	Forward: 5'-TAGGGATCCGATCCCGTGACACGGCC-3'<br>Reverse: 5'-TAGGGTACCCATTGAGAATCTCCTTCTGGG-3'	*BamH*I<br>*Kpn*I
P_{Hsp60}	Forward: 5'-TAGGGATCCGGTGACCACAACGACGCG-3'<br>Reverse: 5'-GCCCTTGCTCACCATGGTACCCATTGCGAAGTGATTCCTCCG-3'	*BamH*I<br>*Kpn*I
P_{L5}	Forward: 5'-TAGAGATCTAGAGGAAACAGCTATGACCATG-3'<br>Reverse: 5'-GCCCTTGCTCACCATGGTACCCATATGCGATCTCCCTTT-3'	*Bgl*II<br>*Kpn*I

a: restriction sites are underline; *b*: gfp-primers used for cloning in the pJH223; *c*: gfp-primers used for cloning in the pLA71.

CC BY
